# Generative Large Language Model—Powered Conversational AI App for Personalized Risk Assessment: Case Study in COVID-19

**DOI:** 10.2196/67363

**Published:** 2025-03-27

**Authors:** Mohammad Amin Roshani, Xiangyu Zhou, Yao Qiang, Srinivasan Suresh, Steven Hicks, Usha Sethuraman, Dongxiao Zhu

**Affiliations:** 1 Department of Computer Science Wayne State University Detroit, MI United States; 2 Department of Computer Science Oakland University Rochester, MI United States; 3 Department of Pediatrics University of Pittsburg Medical Center Children’s Hospital of Pittsburgh Pittsburgh, PA United States; 4 Department of Pediatrics Penn State College of Medicine Hershey, PA United States; 5 Division of Emergency Medicine, Department of Pediatrics Children’s Hospital of Michigan Detroit, MI United States

**Keywords:** personalized risk assessment, large language model, conversational AI, artificial intelligence, COVID-19

## Abstract

**Background:**

Large language models (LLMs) have demonstrated powerful capabilities in natural language tasks and are increasingly being integrated into health care for tasks like disease risk assessment. Traditional machine learning methods rely on structured data and coding, limiting their flexibility in dynamic clinical environments. This study presents a novel approach to disease risk assessment using generative LLMs through conversational artificial intelligence (AI), eliminating the need for programming.

**Objective:**

This study evaluates the use of pretrained generative LLMs, including LLaMA2-7b and Flan-T5-xl, for COVID-19 severity prediction with the goal of enabling a real-time, no-code, risk assessment solution through chatbot-based, question-answering interactions. To contextualize their performance, we compare LLMs with traditional machine learning classifiers, such as logistic regression, extreme gradient boosting (XGBoost), and random forest, which rely on tabular data.

**Methods:**

We fine-tuned LLMs using few-shot natural language examples from a dataset of 393 pediatric patients, developing a mobile app that integrates these models to provide real-time, no-code, COVID-19 severity risk assessment through clinician-patient interaction. The LLMs were compared with traditional classifiers across different experimental settings, using the area under the curve (AUC) as the primary evaluation metric. Feature importance derived from LLM attention layers was also analyzed to enhance interpretability.

**Results:**

Generative LLMs demonstrated strong performance in low-data settings. In zero-shot scenarios, the T0-3b-T model achieved an AUC of 0.75, while other LLMs, such as T0pp(8bit)-T and Flan-T5-xl-T, reached 0.67 and 0.69, respectively. At 2-shot settings, logistic regression and random forest achieved an AUC of 0.57, while Flan-T5-xl-T and T0-3b-T obtained 0.69 and 0.65, respectively. By 32-shot settings, Flan-T5-xl-T reached 0.70, similar to logistic regression (0.69) and random forest (0.68), while XGBoost improved to 0.65. These results illustrate the differences in how generative LLMs and traditional models handle the increasing data availability. LLMs perform well in low-data scenarios, whereas traditional models rely more on structured tabular data and labeled training examples. Furthermore, the mobile app provides real-time, COVID-19 severity assessments and personalized insights through attention-based feature importance, adding value to the clinical interpretation of the results.

**Conclusions:**

Generative LLMs provide a robust alternative to traditional classifiers, particularly in scenarios with limited labeled data. Their ability to handle unstructured inputs and deliver personalized, real-time assessments without coding makes them highly adaptable to clinical settings. This study underscores the potential of LLM-powered conversational artificial intelligence (AI) in health care and encourages further exploration of its use for real-time, disease risk assessment and decision-making support.

## Introduction

### Background

Disease risk assessment is a critical tool in public health surveillance, where demographic variables and social determinants are often used to assess a patient’s susceptibility to disease, predict treatment response, and forecast severity outcomes. Traditionally, these predictions have been carried out using machine learning models trained de novo for each disease or condition using curated tabular data [1-3]. For example, Wang et al [2] developed a linear model–based, multitask learning approach to predict the risk of childhood obesity based on geolocation data. Li et al [3] proposed a mixture neural network to stratify patients and predict heart failure risk within each subgroup.

The advent of transformers has marked a significant shift, allowing researchers to deploy advanced models that improve prediction accuracy and handle complex data structures more effectively. Bidirectional Encoder Representations from Transformers (BERT)–style models [4] have been extensively used in various health care tasks. Notable examples include ClinicalBERT [5] and BioClinicalBERT [6], both trained on clinical notes in the MIMIC-III database. MedBERT [7], further trained on electronic health records (EHRs), achieved a high area under the curve (AUC) scores for disease risk prediction. However, BERT-based models, primarily designed for discriminative tasks, face limitations in processing streaming question-and-answer (QA) pairs typical in conversational data science applications due to their architectural constraints.

### Generative Large Language Models for Health Care

Generative large language models (LLMs), such as OpenAI’s GPT-3 [8], have transcended the limitations of discriminative models by excelling at handling diverse data formats, including both structured clinical data and unstructured text like patient narratives and medical histories. This versatility allows them to integrate and synthesize information from multiple sources, making them highly effective for complex tasks such as predicting disease severity. Generative LLMs have been applied in health care across various domains, including diagnostic support, clinical decision-making, clinical knowledge extraction, and risk prediction with personalized monitoring.

In diagnostic support, generative LLMs like ChatGPT and GPT-4 [9] have been used to aid clinical diagnosis by leveraging structured and unstructured data. Gilson et al [10] assessed ChatGPT’s ability to answer the United States Medical Licensing Examination (USMLE) Step 1 and Step 2 multiple-choice questions, highlighting its potential for medical education and diagnostic assistance. Kung et al [11] evaluated ChatGPT’s clinical reasoning by testing it on structured questions from the USMLE, simulating clinical decision-making tasks without domain-specific training. Ali et al [12] explored the use of ChatGPT to generate patient-friendly clinical letters based on semistructured prompts, aiming to improve communication efficiency while ensuring accessibility for patients. Xv et al [13] used ChatGPT to assist in diagnosing urological diseases using semistructured patient data, demonstrating its potential as a tool for preliminary diagnostic support. Kanjee et al [14] evaluated GPT-4’s diagnostic accuracy in complex clinical cases, showing its ability to generate differential diagnoses based on patient history and clinical findings.

Generative LLMs have also become valuable tools in synthesizing vast amounts of medical literature, enabling clinicians and researchers to stay current with scientific advancements. Tang et al [15] evaluated LLMs in summarizing medical evidence, demonstrating that models like GPT-4 [9] can generate concise summaries of research articles, facilitating faster knowledge assimilation. Sallam [16] discussed how LLMs could assist in systematic reviews and meta-analyses, reducing the effort required in literature search and data extraction.

In risk prediction and personalized patient monitoring, generative LLMs have shown significant potential. Health-LLM [17] integrates wearable sensor data, such as physical activity and heart rate, to predict stress, fatigue, and other health metrics. Leveraging zero-shot learning, the model generalizes effectively across various health prediction tasks without task-specific training. ClinicalMamba [18] excels in analyzing longitudinal EHR notes for disease progression prediction and patient cohort selection by processing unstructured clinical notes over extended sequences.

With increasingly longer context windows, up to 8192 tokens in OpenAI’s GPT-4 [19], generative LLMs can efficiently manage extensive patient records and interaction histories. This capability to process long, varied inputs allows them to generalize effectively even with limited labeled domain-specific data. Furthermore, their ability to handle multiturn conversations positions them uniquely for real-time applications, facilitating no-code disease assessment through interactive patient engagements.

Despite the remarkable performance of proprietary black-box LLMs like GPT-4 and MedPaLM-2 [20], there is growing interest in deploying white-box models in health care and other high-stakes domains. White-box models mitigate risks related to data privacy breaches and hallucination by allowing for full transparency and control over the model’s architecture and parameters. Their smaller size enables deployment on local devices, enhancing data security by keeping sensitive information on the device. Furthermore, the transparent nature of these models facilitates interpretability, which is crucial for explainability in clinical settings.

This shift towards transparent and customizable models is exemplified by PMC-LLaMA [21], adapted from the LLaMA architecture and fine-tuned on extensive health and medical corpora. PMC-LLaMA has outperformed larger models in several health and medical QA benchmarks, highlighting the effectiveness of domain-specific fine-tuning. One of the few studies exploring generative LLMs for disease diagnosis and risk assessment is CPLLM [22]. CPLLM fine-tunes Llama2 [23] as a general LLM and uses BioMedLM [24], a model trained extensively on biological and clinical texts, to perform various prediction tasks, including disease diagnosis and patient outcome forecasting. These models demonstrate the potential of LLMs in understanding complex medical language and reasoning. However, their application to direct disease risk assessment using streaming QA interactions remains limited, and they do not fully leverage the interpretability benefits of white-box models for explainability.

**Figure 1:**
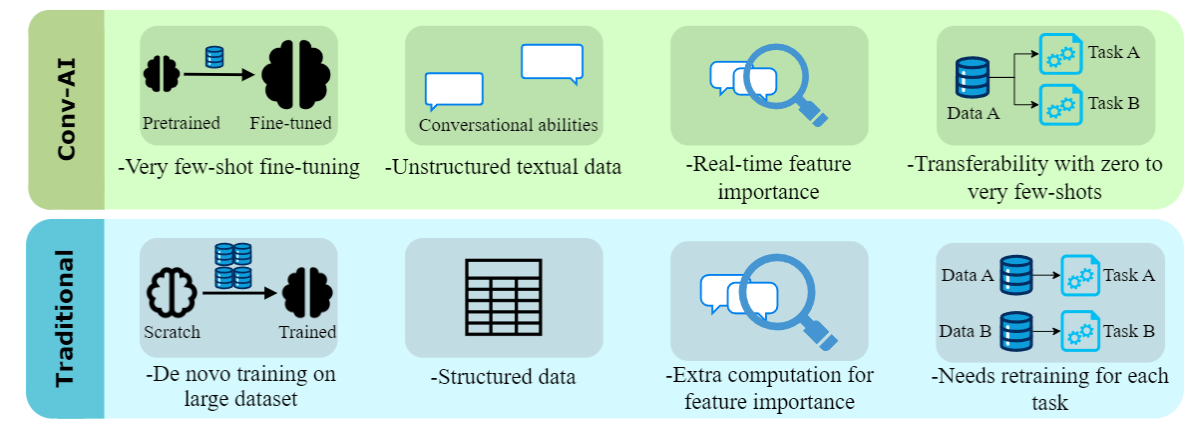
Comparison between large language model (LLM)–based conversational AI (Conv-AI) and traditional machine learning methods for disease risk assessment. The Conv-AI leverages pretrained models that require only very few-shot fine-tuning, can handle unstructured textual data, provide real-time feature importance for each risk assessment it provides, and offer transferability with zero to very few shots for new risk assessment tasks. In contrast, traditional machine learning methods require large datasets for de novo training, process structured data, rely on extra computational steps for instance-specific post hoc feature importance (eg, Shapley additive explanations), and need retraining for each new task.

Our work builds upon these advancements by transitioning from traditional machine learning–based health outcome prediction—which typically relies on structured tabular data—to chatbot-based, no-code prediction using streaming QA interactions. We develop a generative artificial intelligence (GenAI)–powered mobile app that integrates fine-tuned white-box LLMs—including LLaMA2, Flan-T5, and T0 models—as the core for personalized risk assessment and patient-clinician communication. The app provides a natural language interface for risk assessment, processes user responses in real time, and can be deployed locally on devices to enhance data privacy and security. Figure 1 shows a comparison of our work to traditional methods.

### Contributions

Our contributions to the field of LLM-based disease risk assessment are diverse. First and foremost, we transition from traditional machine learning–based health outcome prediction—which typically relies on structured tabular data—to chatbot-based, no-code prediction using streaming QA interactions. This is realized through the development of a GenAI-powered mobile app that integrates fine-tuned LLMs as the core for personalized risk assessment and patient-clinician communication. The app not only assesses disease risk for patients but also provides contextual insights related to risk surveillance and mitigation through natural language conversation.

Second, we demonstrate that generative LLMs can outperform traditional machine learning methods, such as logistic regression [25], random forest [26], and extreme gradient boosting (XGBoost) [27], in low-data regimes, which is critical for medical applications where labeled data are scarce. For instance, our results show that LLMs like the T0-3b model achieve an AUC of 0.75 in zero-shot settings, demonstrating their potential for disease risk assessment even without task-specific training. In addition, we provide a comprehensive comparison of both decoder-only and encoder-decoder models, fine-tuned using the widely adopted, parameter-efficient, low-rank adaptation (LoRA) method [28].

Third, we introduce a feature importance analysis derived from the LLM’s attention layers, providing personalized insights into the most influential factors driving the model’s predictions. This enhances the interpretability and usability of the risk assessment for both patients and clinicians, offering real-time, instance-specific explanations during inference.

## Methods

### Our Research Objective

**Figure 2:**
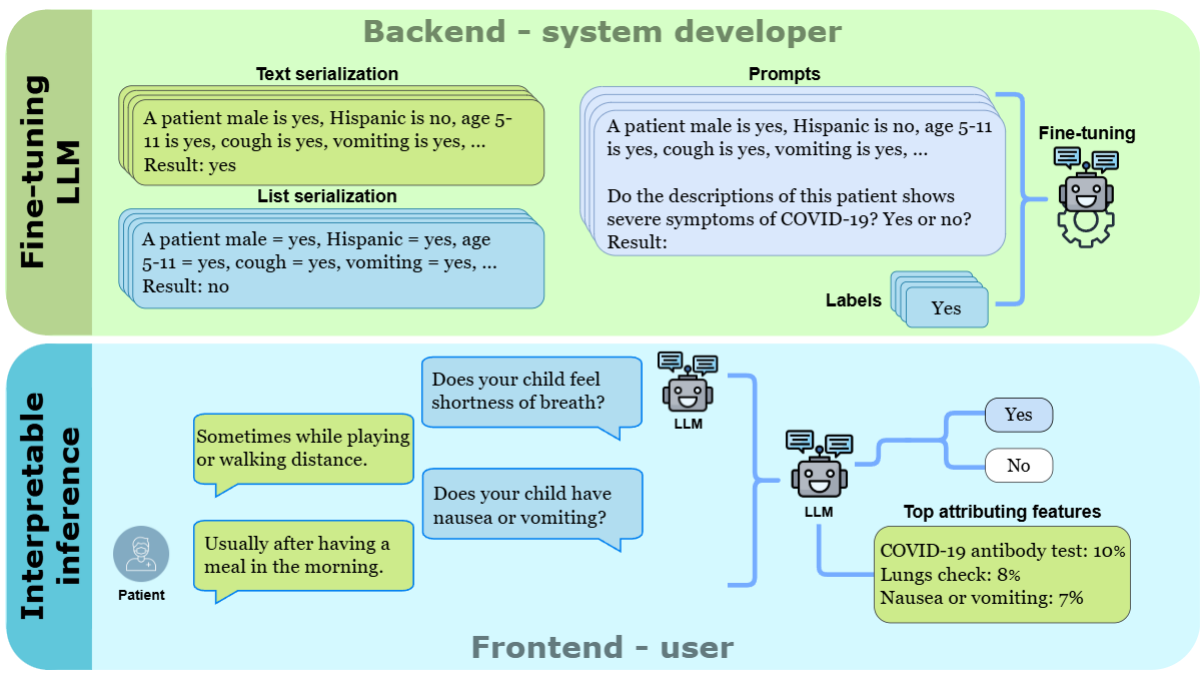
Workflow for few-shot COVID-19 severity risk assessment using generative large language models (LLMs) with different serialization techniques. The top section, labeled "Backend - system developer," shows the fine-tuning phase where a few-shot sample of patient data, serialized through list and text templates, is used to fine-tune the LLMs. This backend process includes the creation of prompts and corresponding labels for model fine-tuning. The bottom section, labeled "Frontend - user," illustrates how a conversational chatbot interacts with users through our application to gather responses through streaming QA interactions. These responses are analyzed by the fine-tuned LLM in real time, providing risk assessments and highlighting the top attributing features that explain the model’s risk assessment. QA: question-and-answer.

The primary objective of this study is to explore the effectiveness of pretrained generative LLMs in no-code risk assessment of disease severity using few-shot multihop QA interactions. We aim to evaluate how these generative LLM-powered chatbots can use streaming QA interactions to accurately classify patient outcomes as severe or nonsevere, which is crucial for early risk assessment and optimizing health care resource allocation. Through a case study of COVID-19 severity risk assessment, we developed an app that uses open-source generative LLMs to determine the severity of COVID-19 outcomes. This involves leveraging the models’ capabilities in zero-shot and few-shot settings, with a focus on the use of serialization techniques to enhance their effectiveness and generalizability. We also integrate real-time feature importance to provide interpretable risk assessments. Figure 2 shows the workflow of our approach, from fine-tuning generative LLMs using serialized QA pairs to real-time risk assessment through a conversational interface.

### Data Collection

**Figure 3:**
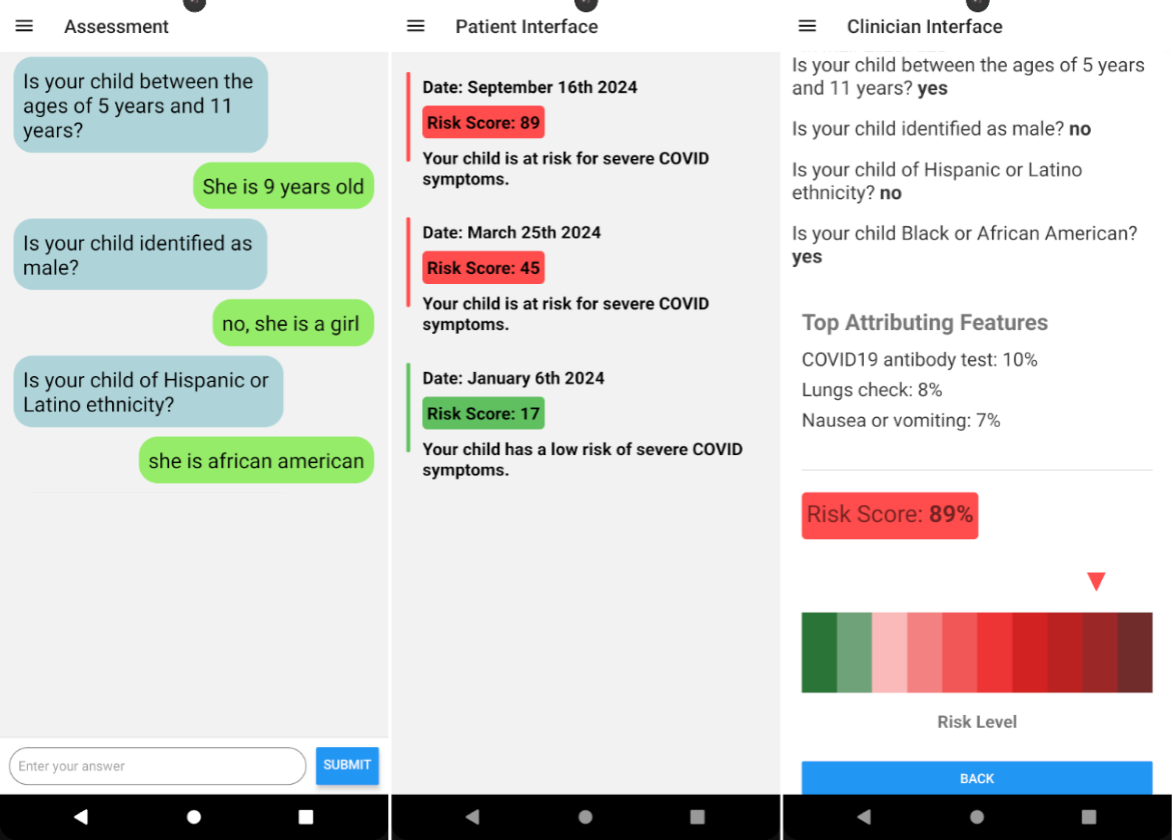
Overview of our mobile app design, showcasing patient data collection, real-time risk assessment using large language models (LLMs), and clinician review interface.

A dataset was collected from the emergency departments of Children’s Hospital of Michigan and UPMC Children’s Hospital of Pittsburgh between March 2021 and February 2022. Table 1 provides an overview of the binary features used in our study, including demographic, clinical, and social determinants that may influence COVID-19 severity risk. The dataset includes a total of 393 participant records, each characterized by responses to a series of carefully designed questions (see Figure 3 for sample QA pairs).

The severity of illness was defined based on the presence of any of the following criteria:

1. Requirement for supplemental oxygen (≥50% fraction of inspired oxygen)
2. Need for mechanical ventilation or noninvasive positive pressure ventilation (bilevel positive airway pressure and continuous positive airway pressure)
3. Need for vasopressors or inotropes
4. Requirement for extracorporeal membrane oxygenation
5. Cardiopulmonary resuscitation
6. Death from a related cause within 4 weeks after discharge

Children meeting any of these criteria were categorized as having severe illness. These outcomes were determined through chart reviews and parent surveys conducted 30 days after discharge [29].

Outliers were removed, and feature selection was performed using Shapley additive explanations values [30], resulting in the final dataset used for analysis.

**Table 1:** Binary features used in the study. The dataset consists of 393 patient records with 15 features representing demographics, clinical symptoms, and social determinants. These features serve as inputs for traditional machine learning models and are also serialized for fine-tuning generative large language models (LLMs).

Feature and label		Count, n
**f1. Ages 5 to 11 years**		
	No	294
	Yes	99
**f2. Gender**		
	Female	332
	Male	61
**f3. Hispanic**		
	No	359
	Yes	34
**f4. African American**		
	No	215
	Yes	178
**f5. Service at stores**		
	Good	335
	Poor	78
**f6. Insurance**		
	No	387
	Yes	6
**f7. Headache**		
	No	332
	Yes	61
**f8. Fever**		
	No	211
	Yes	182
**f9. Cough**		
	No	210
	Yes	183
**f10. Shortness of breath**		
	No	292
	Yes	101
**f11. Exposed to COVID-19 individuals**		
	No	343
	Yes	50
**f12. Nausea or vomiting**		
	No	272
	Yes	121
**f13. Lungs check**		
	Bad	317
	Good	76
**f14. Eye redness**		
	No	381
	Yes	12
**f15. COVID-19 antibody test**		
	Negative	364
	Positive	29
**f16. Outcome (severity)**		
	No	284
	Yes	109

### Tabular Data for Traditional Models

As traditional machine learning methods require tabular data as input, we formalize the questionnaire QA pairs 
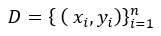
, where *n*=393, 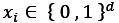 represents the binary feature vector of the *i*-th instance where *d*=15, and 
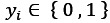
 denotes the binary class label indicating the presence or absence of severe COVID-19 symptoms determined by clinicians.

Each feature vector *x_i_* consists of binary indicators representing social determinants and clinical and demographic factors that may influence the severity of COVID-19, such as age, preexisting conditions, vital signs, and laboratory test results. These features are shown in Table 1. The feature names are denoted as 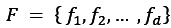, where each *f_j_* is a natural-language string describing the corresponding attribute.

The task is to predict the binary outcome *y_i_* based on the information provided in *x_i_*. This constitutes a supervised learning problem where the objective is to train a model to minimize prediction error on unseen data.

### Serialization for New Conversational AI

At the time of data collection from 2021 to 2022, we did not yet have a chatbot for automated data donations from users, so we used a questionnaire to collect answers from each patient based on a set of questions designed for this study. As a result, the native format of the dataset consists of QA pairs, which were subsequently serialized to fine-tune the generative LLMs for the risk assessment task. It is important to note that the fine-tuned model is capable of assessing risk using streaming QA interactions in real time (Figures 2 and 3).

To achieve serialization, the features in our dataset are denoted as 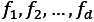, and their associated values as 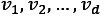. This notation provides a structure that is transformed into natural language prompts for the LLM.

We used two main serialization methods from TABLLM [31], the list template and the text template, to create natural language representations of the data. As shown in Figure 2, the list template links each feature with its value using an equal sign (“=”), while the text template uses a narrative structure with the word “is” to connect each feature with its value. These templates enable us to evaluate which serialization approach better translates the data into actionable insights by the LLM.

### Generative LLMs

We explore the capabilities of 3 white-box LLMs—LLaMA2 [23], T0 [32], and Flan-T5 [33]—focusing on their application in risk prediction for COVID-19 using both the native QA pairs and the formatted tabular dataset.

To our knowledge, this is one of the first attempts leveraging generative LLMs and conversational data science for disease risk assessment across various LLMs and few-shot settings. Our selection includes both decoder-only (LLaMA2) and encoder-decoder architectures (T0 and Flan-T5), allowing for a comprehensive assessment and comparison of their performance. The white-box nature of these models is particularly advantageous as it enables setup on local hosts with private datasets, ensuring precise risk assessment by allowing direct access to model weights and logits.

The input to the LLMs is a serialized string generated from the tabular data using the previously explained serialization strategies. Given a feature vector 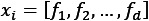. and their associated values 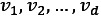, the serialized input string *S_i_* can be represented using either the list template or text template serialization methods (Figure 2).

These feature vectors originate from the structured dataset described in Table 1, which provides the foundation for both traditional and generative model comparisons.

The LLM processes the serialized input string *S_i_* and outputs logits for the next token in the sequence. We focus on the logits corresponding to the tokens “yes” and “no,” which indicate severe or nonsevere symptoms, respectively. The probabilities for these tokens are obtained by applying the softmax function to the logits:



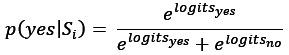



The probability 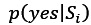 indicates the likelihood of severe symptoms based on the input data *S_i_*. This probability is directly used as the severity risk score for evaluation purposes.

To determine the binary predicted label 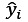 from this probability:



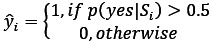



The probability score 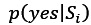, reflecting the severity risk, is used to compute the AUC for evaluation (Figure 2).

### Evaluation Setting

#### Zero-Shot Setting

In the zero-shot setting, our approach leverages the intrinsic capabilities of LLMs. These models, unlike traditional classifiers such as logistic regression and XGBoost, have been extensively pretrained on diverse datasets. This extensive pretraining enables them to apply their accumulated world knowledge directly to specific classification tasks without additional training, demonstrating exceptional generalizability.

We assess the zero-shot prediction effectiveness of these LLMs by presenting them with tasks aligned with our study’s objectives that they have not been specifically trained on. The models interpret and classify new, unseen data solely based on their pretrained knowledge. This approach not only highlights the potential of LLMs in real-world applications but also evaluates their ability to generalize from their training to novel scenarios in healthcare.

This zero-shot methodology allows us to evaluate how well these LLMs can recognize and classify complex, previously unseen patterns in health care data, providing valuable insights into their practical applicability and limitations in clinical settings.

#### Few-Shot Fine-Tuning

In the few-shot setting, we use sample sizes of 2, 4, 8, 16, and 32 to fine-tune the LLMs, aiming to examine the effect of training sample size on model performance compared to traditional classifiers. To ensure fairness and reduce bias in the fine-tuning process, we maintain a balanced ratio of positive 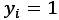 and negative 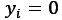 samples, with an equal number of examples from each class in each sample size.

To enhance computational efficiency in adapting the LLMs to our specific tasks, we employ a parameter-efficient fine-tuning approach using LoRA [27]. Instead of adjusting all parameters within the model, LoRA involves training a small proportion of parameters by integrating trainable low-rank matrices into each layer of the pretrained model. This method allows the model to quickly adapt to new tasks by optimizing only a subset of parameters, thereby preserving the general capabilities of the LLM while enhancing its performance on task-specific features.

### Feature Importance Analysis

In disease risk assessment, interpretability is as critical as accuracy, particularly when both are provided to the user in real time. Here, we introduce a novel approach for analyzing feature importance by leveraging the attention mechanisms inherent in the output layers of generative LLMs. This method provides additional insights into the risk assessment process of the model, which is valuable for both clinicians and patients in understanding the factors contributing to the model's output.

Our approach involves extracting attention scores from the model’s output layer, where the attention assigned to each input token is interpreted as an indicator of feature importance. We compute the attention for each feature-value pair and associate the average attention score with the corresponding feature. This provides a holistic view of which features, along with their associated values, influence the model’s output.

**Figure 4:**
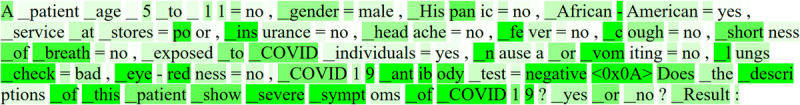
The attention map for a predicted positive case where the darker color represents larger attention weights for each token. The prompts are tokenized to mimic the actual inputs to the large language models (LLMs).

In Figure 4, the attention map illustrates the attention scores for a predicted positive case by the LLM, where darker shades represent higher attention scores assigned to specific feature-value pairs.

For an input sequence such as:

*A patient* 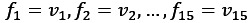

Do the descriptions of this patient show severe symptoms of COVID-19? Yes or no? Result:

We calculate attention scores for each feature-value pair in the original sequence. The average attention score for each feature-value pair is then computed, and the score is associated with the feature itself, offering a representation of feature importance in the context of disease severity risk. As shown in Figure 4, any missing data in both the training and inference stages could be handled by having the value as “none” and having the model make the prediction; this will impact the prediction depending on the feature missing, but the free-text input of the LLMs still allows for a prediction to happen.

This normalized attention score serves as a proxy for feature importance, offering clinicians and patients a clearer understanding of which features (eg, age, preexisting conditions, vital signs, etc) are most influential in the model's assessment of COVID-19 severity risk. As illustrated in Multimedia Appendix 1, the plot shows the normalized attention scores from the LLaMA2-7b model in the 32-shot setting for two test cases: one positive (yes) and one negative (no).

For the positive case, the top five features with the highest attention scores, as shown in this figure, are:

1. f15: COVID-19 antibody test
2. f13: Lungs check
3. f12: Nausea or vomiting
4. f9: Cough
5. f14: Eye redness

By integrating this analysis into our mobile app, we enhance the interpretability of LLM-based risk assessments, empowering users with deeper insights into the model's reasoning process.

### Mobile App

To provide users with code-free disease severity risk assessment and enhance user experience, we developed a mobile chatbot powered by the aforementioned generative LLMs. This app is designed to facilitate the assessment and management of COVID-19 in children, with potential applicability to other diseases and conditions. It offers two versions: one for patients to donate their health information via answering the questions and receiving real-time severity risk assessments, and another for clinicians to manage, review, and interpret the sessions donated by patients. The primary goals are to enhance early detection of severe outcomes, improve patient-clinician communication, and streamline the overall risk assessment process.

The app targets patients, clinicians, and other health care providers involved in managing preclinical cases. It leverages the capabilities of generative LLMs to analyze patient responses and provide immediate feedback on the risk of severe symptoms. Developed using React Native and JavaScript for the front end, Firebase for database management, and various frontend technologies, the app provides a user-friendly, efficient, and effective solution for managing disease risks. It aims to improve patient outcomes by facilitating timely and informed decision-making.

#### Database Structure

Our mobile app uses Firebase for database management, structured into three primary collections: Users, Questions, and Answers.

**Figure 5:**
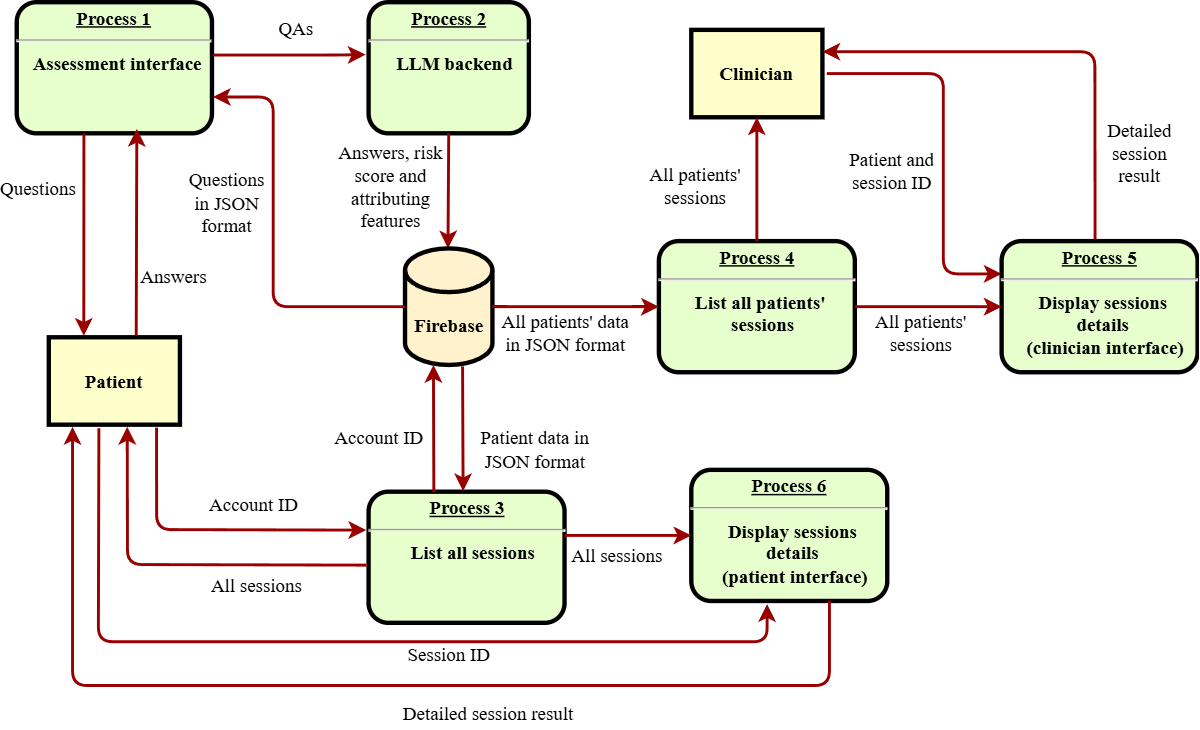
Data flow diagram where we map out the flow of information between different processes of large language model (LLM) backend, Firebase, and mobile app interfaces for both patient and clinician.

The data flow between the patient, LLM backend, Firebase, and interfaces for both patients and clinicians is illustrated in Figure 5. This figure highlights the interactions among processes, including the assessment submission, session management, and result retrieval.

- Users: This collection includes essential user information such as ID, Email, and isAdmin. The ID uniquely identifies each user, the Email serves as contact information, and the isAdmin field (Boolean) indicates whether the user has administrative privileges (clinicians) or not (patients).
- Questions: Each document in this collection has a unique ID and a Description field. The ID is used to reference questions in the Answers collection, and the Description contains the text of the question posed to the user, ensuring clarity and specificity in data mapping.
- Answers: This collection records user responses during their sessions. Each document includes a session ID and an array of answers where each entry links to the relevant Question ID from the Questions collection. In addition, it contains a Text field for the user's detailed response; an Answer field for the LLM-generated response (eg, yes or no); a Date field marking the session's completion time; a Risk Score field, which is derived from the user’s responses and utilized for subsequent risk prediction by the LLM; and an Important Features field, which stores the key features identified by the LLM’s attention scores that contributed to the risk assessment.

#### User Interface: Assessment

**Figure 6:**
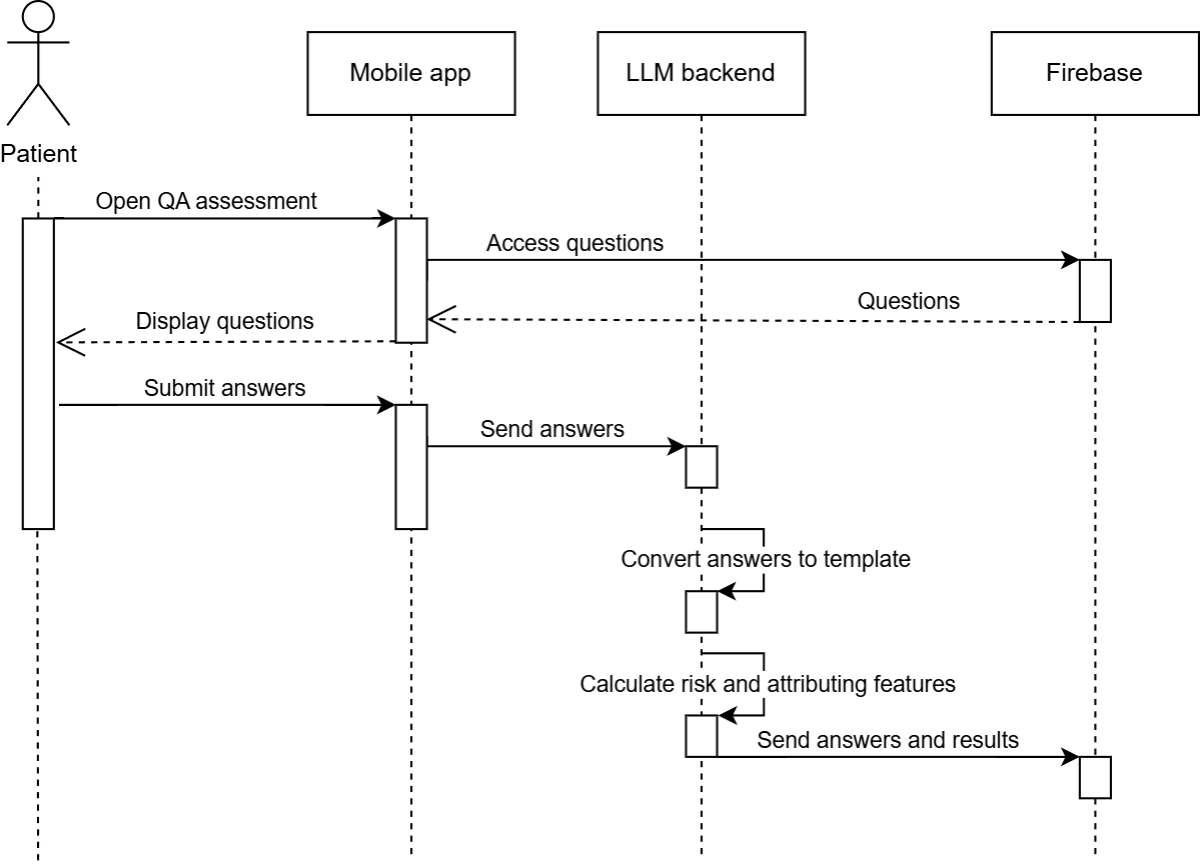
Sequence diagram for the Assessment page, where the patient takes the risk assessment and the large language model (LLM) backend calculates the results, which will be saved to the Firebase. QA: question-and-answer.

The step-by-step workflow for conducting an assessment and storing results in Firebase is detailed in Figure 6. This sequence diagram outlines the interaction between the patient, mobile app, LLM backend, and database.

As shown in Figure 3, on the Assessment page, we leverage the power of LLMs to engage in a conversation with the patient. This interaction allows us to ask questions and gather contextual information for each response. By doing so, we retrieve a binary answer (yes or no) using the LLM, which is then provided to the primary care physician along with the patient’s context to aid in decision-making.

After the user responds to each question, we use our LLM to generate a binary answer. This involves providing the LLM with instructions that include the question and the user’s response and asking the LLM to interpret the response into a binary answer (yes or no). This sequential process is performed for all questions. Currently, the input for the final LLM-based risk assessment, which predicts the COVID-19 severity risk, is based solely on the set of binary answers generated by the LLM. Future enhancements could incorporate the original user responses to improve context understanding.

We currently use the Llama2-7b application programming interface (API) for answer retrieval. Our long-term goal is to integrate a fine-tuned LLM hosted on our servers to ensure better optimization and accuracy specific to our dataset, as evidenced by the improved performance results discussed in this paper.

#### User Interface: Patient and Clinician Results

**Figure 7:**
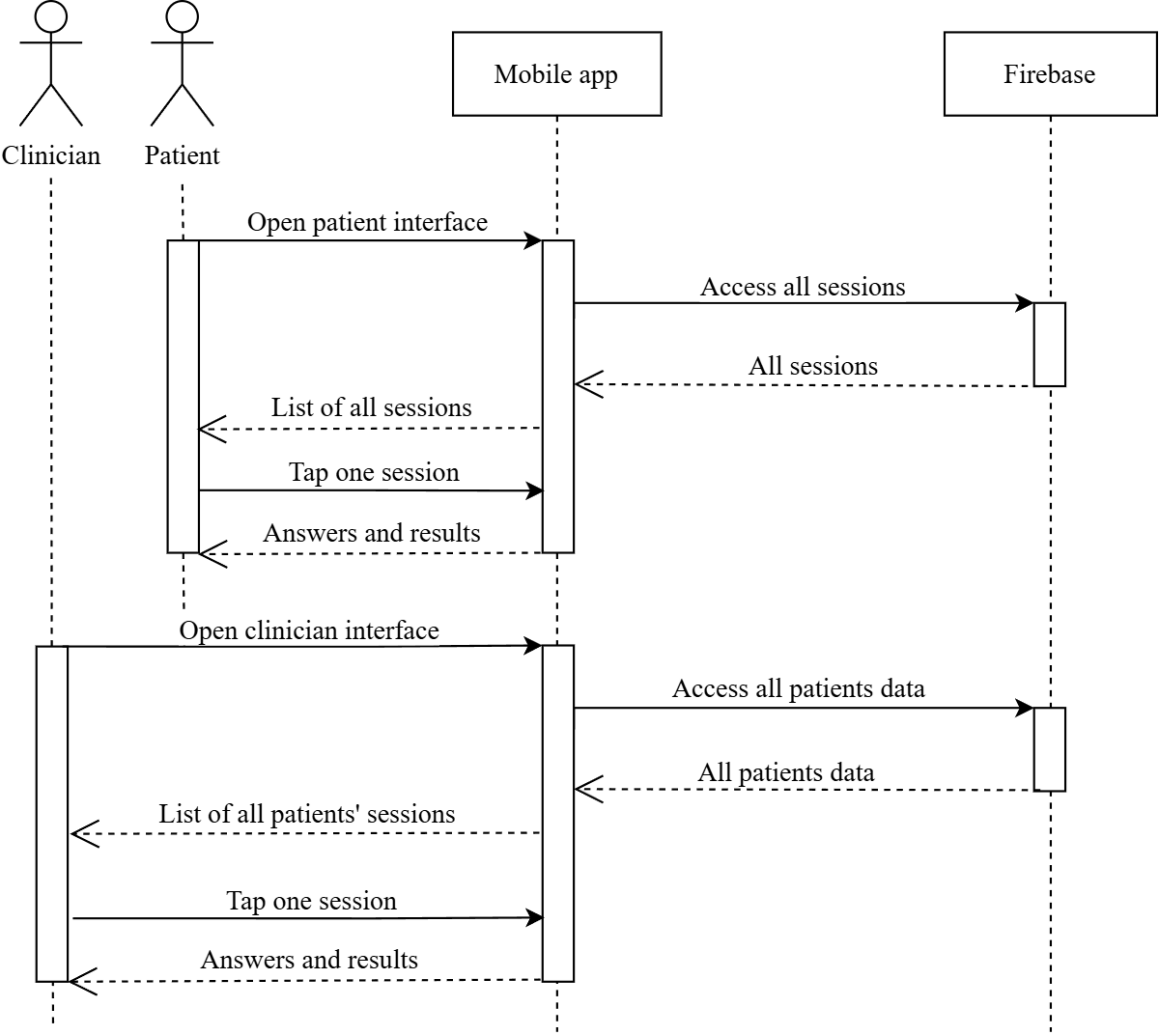
Sequence diagram for displaying patients’ session results. As shown, each patient has access to all their own sessions while the clinician can access all patients’ sessions.

Figure 7 illustrates the interaction flows for both patients and clinicians as they access session details and results. This sequence diagram shows how patient data and assessments are retrieved and displayed in real time.

Patients can submit a session at any time, receiving an immediate risk assessment in the Patient Interface section (Figure 3). This section displays all sessions submitted by the current user, along with their respective risk assessments.

In the Clinician Interface section, clinicians can access all sessions from their patients, organized by patient ID, for efficient review. Each session includes a comprehensive report featuring the predicted risk score, ensuring transparency and aiding in clinical decision-making.

Upon submission, a patient’s session is instantly available in both the patient’s and clinician’s panels. While patients can only view their own sessions, clinicians can review all sessions from their assigned patients. This setup supports real-time updates through Firebase, facilitating seamless communication and follow-up between patients and their health care providers. Furthermore, the app provides personalized feature importance analysis based on the LLM’s attention layers, giving both patients and clinicians additional insights into the most critical factors influencing the risk assessment.

### Ethical Considerations

The data collected and used for this study were approved by the University of Pittsburgh Institutional Review Board (MOD21010046-003; approval date: February 25, 2021). Informed consent was obtained from all legal caregivers, and when age appropriate, an informed assent was also obtained from the participants. Before the use of this study, the data were subject to a multistep anonymization procedure with personally identifying information marked and deleted.

## Results

### Training and Fine-Tuning Settings

In our experiments, we used a rigorous hyperparameter tuning strategy to optimize model performance, supported by a robust setup to ensure diverse dataset initialization and minimize potential biases. For both traditional machine learning methods and LLMs, we used 5 specific random seeds—0, 1, 32, 42, and 1024—to create diverse dataset splits. The dataset of 393 samples was divided into 256 training, 59 validation, and 78 testing segments, preserving a consistent positive-to-negative ratio of approximately 0.38.

For both traditional methods and LLMs, training was conducted using up to 32 shots to evaluate performance in the few-shot regime. For few-shot settings ranging from 2 to 32 shots, we ensured a balanced sampling of positive and negative examples in the training set, maintaining an equal number of instances from each class to avoid biases during training. Key hyperparameters, such as the learning rate, were optimized using grid search, with the learning rate set to 3 x 10^–4^. The batch size matched the number of shots, and training consistently ran for 128 epochs to ensure convergence. During fine-tuning with LoRA, validation loss was monitored to select the best model checkpoint, minimizing overfitting and enhancing generalization to the test set. The optimization used cross-entropy loss, aligning with the binary classification task of predicting COVID-19 severity. This comprehensive setup ensured robust and interpretable model performance, particularly in low-data settings.

### Effects of Serialization

#### Overview

Table 2 shows the performance of different serialization methods for the LLMs across various few-shot settings. We evaluated 2 primary serialization methods: list template and text template, across models tested with 0, 2, 4, 8, 16, and 32 training shots to observe performance variations with the number of training examples.

The list template often exhibited better performance at lower shot counts, while the text template typically outperformed the list template as the number of training examples increased. The following summarizes the performance trends for each model.

**Table 2:** Performance of models across different shot settings. All values represent the average area under the curve (AUC) across 5 random seeds rounded to 2 decimal places. In addition, SDs given across the 5 random seeds are shown. The suffixes “-L” and “-T” represent list serialization and text serialization, respectively.

Model	Number of shots					
	0, AUC^a^ (SD)	2, AUC (SD)	4, AUC (SD)	8, AUC (SD)	16, AUC (SD)	32, AUC (SD)
Llama2-7b-L	0.54 (.05)	0.69 (.07)	0.69 (.06)	0.68 (.04)	0.63 (.04)	0.66 (.07)
Flan-t5-xl-L	0.62 (.03)	0.64 (.02)	0.63 (.02)	0.68 (.06)	0.66 (.05)	0.69 (.06)
Flan-t5-xxl-L	0.60 (.03)	0.61 (.03)	0.61 (.05)	0.62 (.06)	0.59 (.10)	0.65 (.11)
T0pp(8bit)-L	0.69 (.04)	0.70 (.07)	0.70 (.05)	0.70 (.05)	0.68 (.06)	0.70 (.10)
T0-3b-L	0.68 (.04)	0.67 (.04)	0.68 (.05)	0.70 (.04)	0.67 (.04)	0.67 (.07)
Llama2-7b-T	0.59 (.05)	0.69 (.03)	0.69 (.01)	0.64 (.07)	0.63 (.05)	0.67 (.06)
Flan-t5-xl-T	0.69 (.03)	0.69 (.02)	0.69 (.03)	0.71 (.05)	0.69 (.04)	0.70 (.05)
Flan-t5-xxl-T	0.61 (.04)	0.58 (.03)	0.63 (.08)	0.59 (.10)	0.62 (.09)	0.63 (.10)
T0pp(8bit)-T	0.67 (.02)	0.65 (.05)	0.66 (.05)	0.68 (.04)	0.65 (.08)	0.67 (.08)
T0-3b-T	0.75 (.04)	0.65 (.06)	0.65 (.05)	0.68 (.03)	0.67 (.04)	0.65 (.08)
Logistic regression	—^b^	0.57 (.07)	0.55 (.10)	0.64 (.06)	0.61 (.11)	0.69 (.08)
Random forest	—	0.57 (.07)	0.57 (.06)	0.62 (.08)	0.66 (.07)	0.68 (.07)
XGBoost^c^	—	0.50 (.00)	0.50 (.00)	0.50 (.00)	0.54 (.06)	0.65 (.03)

^a^Average area under the curve.

^b^Not applicable.

^c^XGBoost: extreme gradient boosting.

#### Llama2-7b

In the zero-shot setting, the text template achieved an AUC of 0.59 compared to 0.54 for the list template. At 2 training shots, both templates achieved an AUC of 0.69, but the text template began to outperform, reaching an AUC of 0.67 at 32 training shots compared with 0.66 for the list template.

#### Flan-t5-xl

The text template consistently outperformed the list template across most shot settings. At 2 training shots, the text template achieved an AUC of 0.69 compared to 0.64 for the list template, and this lead continued up to 32 shots, where the text template achieved an AUC of 0.70 compared to 0.69 for the list template.

#### Flan-t5-xxl

Both templates showed similar performance in the early few-shot settings. At 2 training shots, the list template achieved an AUC of 0.61, slightly outperforming the text template, which achieved an AUC of 0.58. By 32 training shots, the list template achieved an AUC of 0.65, slightly outperforming the text template, which achieved an AUC of 0.63.

#### T0pp (8bit)

In the zero-shot setting, the list template led with an AUC of 0.69 compared to 0.67 for the text template. This lead was maintained through most shot settings, with both templates achieving around 0.70 AUC by 32 shots.

#### T0-3b

The text template outperformed the list template in the zero-shot setting, achieving an AUC of 0.75 compared to 0.68 for the list template. In the 2-shot setting, the list template performed slightly better, with an AUC of 0.67 compared to 0.65 for the text template. At 32 shots, the text template closed the gap with an AUC of 0.65 compared with 0.67 for the list template.

In Table 3, we can also compare the best-performing models across different shots, constraining the recall to be higher than 0.8. This gives us better insights into their performance in population screening for early health risks, where recall is considered more important than precision.

Overall, while the list template often provides an initial advantage in early few-shot settings, the text template shows competitive performance as the number of training examples increases. This suggests that serialization choice can be important in low-data regimes. The text template’s strong performance in the zero-shot setting, particularly for the T0-3b model, highlights its potential when no training data is available.

**Table 3:** Precision, recall, and F1-score of the best performing models across different shots averaged over 5 random seeds.

Shot	Best model	Threshold	Precision	Recall	*F*_1_-score
0	T0-3b	0.04	0.37	0.85	0.52
2	T0pp	0.12	0.34	0.81	0.46
4	T0pp	0.24	0.35	0.83	0.49
8	flan-t5-xl	0.17	0.38	0.80	0.50
16	flan-t5-xl	0.15	0.34	0.85	0.48
32	flan-t5-xl	0.16	0.36	0.81	0.49

### LLMs Versus Traditional Machine Learning Methods

Our study highlights the versatility of LLMs for various health care apps, particularly in scenarios with limited data. To benchmark their performance against traditional machine learning methods, we compared LLMs with logistic regression, random forest, and XGBoost.

LLMs benefit from extensive pretraining, allowing them to generalize well to “unseen” data, unlike traditional methods that require substantial amounts of training data. As shown in Table 2, LLMs like T0-3b-T achieved an AUC of 0.75 in the zero-shot setting, demonstrating a good performance even without task-specific fine-tuning. This demonstrates the effectiveness of LLM-powered risk assessment without the need for additional labeled data.

**Figure 8:**
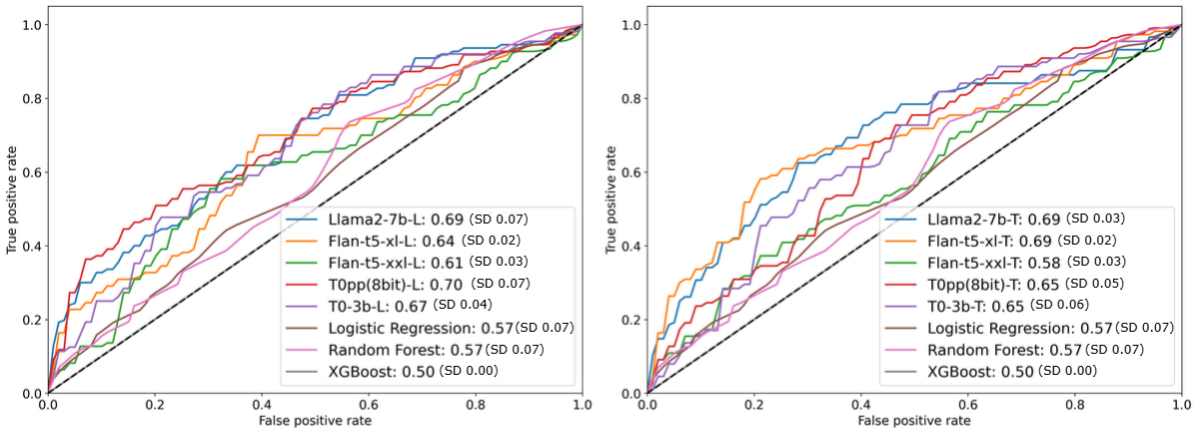
Average area under the curve (AUC) in a 2-shot setting over 5 different seeds. The left panel shows results using the list serialization (-L) approach, while the right panel shows results using the text serialization (-T) approach. XGBoost: extreme gradient boosting.

In the 2-shot setting, LLMs continue to show strong performance relative to traditional methods. For instance, Figure 8 compares the average AUC across 5 different seeds in this scenario. The left panel shows results using the list serialization (-L) approach, while the right panel shows results using the text serialization (-T) approach. In this 2-shot scenario, LLMs such as T0pp(8bit)-L and Flan-t5-xl-T achieve AUCs of 0.70 and 0.69, respectively, clearly outperforming traditional methods, including logistic regression, random forest, and XGBoost, which achieved AUCs of 0.57, 0.57, and 0.50, respectively.

LLMs’ ability to perform well with minimal data highlights their advantage in low-data regimes. This makes them particularly suitable for real-time, no-code health care apps where rapid decision-making is required, even in scenarios where labeled data is scarce.

Furthermore, LLMs’ capacity to handle streaming data formats, such as multihop QA pairs, enhances their integration into conversational interfaces, supporting real-time patient-clinician interactions. This flexibility offers significant usability in clinical settings where personalized and immediate risk assessments are needed (Figure 1).

Overall, while traditional methods may improve with larger datasets, LLMs demonstrate a clear advantage in dynamic, low-data health care environments. Their ability to handle incomplete data and streaming input formats makes them robust for real-world applications requiring adaptability and speed.

## Discussion

### Principal Findings

Our research demonstrates that generative LLMs provide a robust and no-code approach for predicting COVID-19 severity, which is particularly effective in low-data regimes. These models excel in zero-shot and few-shot settings, showcasing their ability to perform well without extensive domain-specific training. This is crucial for real-time applications requiring immediate and reliable predictions, highlighting their exceptional generalizability compared with traditional classifiers like logistic regression, random forest, and XGBoost, which typically require more labeled data to achieve comparable performance.

Generative LLMs effectively handle diverse input formats, integrating both structured clinical data and unstructured natural language inputs from patient interactions. This flexibility enables them to synthesize information from various sources, such as patient medical histories and symptom descriptions, enhancing their usability in dynamic health care settings. In our study, we incorporated these models into a conversational interface, which facilitates real-time patient-clinician interactions and immediate risk assessments. This setup supports continuous data collection and leverages the conversational capabilities of LLMs to optimize clinical decision-making and resource allocation.

### Future Directions and Limitations

Future work should focus on integrating continuous clinician-patient conversational data for fine-tuning or in-context learning, extending the application of LLMs beyond static disease prediction models. Techniques like chain of thought and chain of interaction, which align with the interactive nature of medical consultations, show promise for enhancing model performance in interpreting and responding to patient data in real-time settings. While our study used models like T0pp with parameter-efficient fine-tuning using LoRA, future research could explore newer and more advanced small language models such as LLaMA3-8b and Mistral-7b-Instruct, which have demonstrated exceptional performance in low-data regimes. These models could offer greater efficiency and accuracy as computational resources and methodologies advance, supporting more sophisticated and scalable applications in health care [34,35].

However, limitations remain that warrant further exploration. This study does not address the critical issue of handling sensitive data, such as personally identifiable information (PII), within health care datasets. Incorporating a dual dataset that includes both PII and non-PII data could facilitate machine unlearning research, allowing models to selectively forget sensitive information while retaining predictive capabilities from nonsensitive data. This would ensure compliance with privacy regulations and enhance the ethical deployment of LLMs in health care. Advancing privacy-preserving techniques, such as selective forgetting mechanisms, would not only safeguard sensitive data but also support broader trust in the use of LLMs in clinical settings.

As these models evolve, vulnerabilities such as adversarial attacks during in-context learning pose significant risks. Studies have shown that manipulated inputs can lead to inaccurate or harmful predictions, particularly in high-stakes tasks like health care risk assessment [36]. Addressing these risks is crucial to ensure that LLMs remain reliable and safe for broader adoption in health care applications. Enhanced resilience to adversarial techniques, combined with privacy-preserving methods, will be key to building robust and trustworthy systems. By addressing these challenges, future research can ensure that LLMs not only deliver accurate predictions but also adhere to ethical and privacy standards in real-world settings.

### Conclusions

In conclusion, generative LLMs offer a valuable tool for no-code risk assessment in low-data regimes. Their ability to perform zero-shot or few-shot transferability to new diseases or conditions and handle complex, varied inputs positions them as key assets for enhancing health care interventions and resource management. Furthermore, the incorporation of feature importance analysis derived from the LLM’s attention layers provides an additional layer of interpretability, offering personalized insights into the decision-making process for both patients and clinicians.

## Appendix

Multimedia Appendix 1 Normalized attention scores from LLaMA2-7b in the 32-shot setting, showing feature importance for 2 test cases, 1 positive (yes) and 1 negative (no), simultaneously with the risk assessment.

## Abbreviations

AI: artificial intelligence
API: application programming interface
AUC: area under the curve
BERT: Bidirectional Encoder Representations from Transformers
EHR: electrical health record
GenAI: generative artificial intelligence
LLM: large language model
LoRA: low-rank adaptation
PII: personally identifiable information
QA: question-and-answer
SHAP: Shapley additive explanations
USMLE: United States Medical Licensing Examination
XGBoost: extreme gradient boosting
