# Supplementary figures and images for: Generative Large Language Model—Powered Conversational AI App for Personalized Risk Assessment: Case Study in COVID-19
Source: JMIR AI. 2025 Mar 27;4:e67363. doi: 10.2196/67363 (PMC11986386; doi:10.2196/67363)

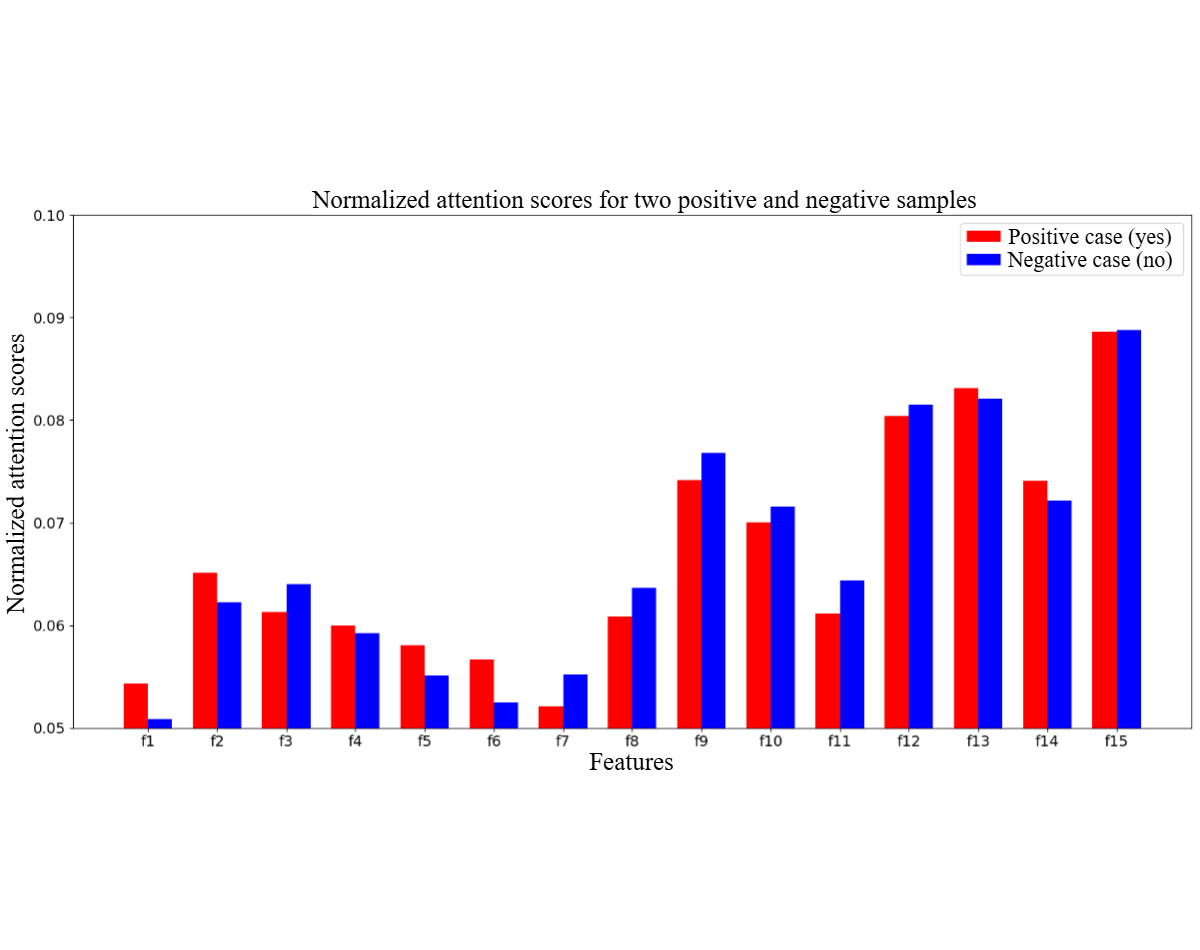

Supplement: Multimedia Appendix 1 [file ai_v4i1e67363_app1.png]
